# Improving regioselectivity of phenylalanine aminomutase from *Taxus chinensis* by semi-rational mutagenesis for the biocatalytic amination of cinnamates

**DOI:** 10.3389/fbioe.2024.1417962

**Published:** 2024-08-22

**Authors:** Tao Tang, Miao Wang, Yunyun Zhang, Yijun Chen

**Affiliations:** Laboratory of Chemical Biology and State Key Laboratory of Natural Medicines, China Pharmaceutical University, Nanjing, Jiangsu, China

**Keywords:** biocatalysis, enzyme engineering, phenylalanine aminomutase, regioselectivity, phenylalanine derivatives

## Abstract

The occupancy of the binding pocket by the substrate ultimately determines the outcome of enzyme catalysis. Previous engineering and substrate scope of phenylalanine aminomutase from *Taxus chinensis* (TcPAM) has generated valuable knowledge about the regioselectivity with biocatalytic potentials for the preparation of α- and β-phenylalanine and their derivatives. However, the significantly different regioselectivity during the amination of cinnamates by TcPAM is not fully understood. In this study, we take a reconstruction approach to change the whole binding pocket of TcPAM for probing the factors affecting the regioselectivity, resulting in variant C107S/Q319M/I431V reaching a 25.5-fold enhancement of the β/α product ratio toward *trans*-cinnamate acid. Furthermore, when substituted cinnamates were used as substrates, the regioselectivity was strongly correlated with various changes in the binding pocket, and value-added 2-Cl-α-Phe (100% α-selectivity) and 4-CH_3_-β-Phe (98% β-selectivity) were individually verified by the mutants L104A and Q319M at a preparative scale, exemplifying the application feasibility of our engineering strategy. The present study uncovered the cooperative connection between aromatic binding and carboxylate binding to affect the regioselectivity, which provides new insights into the determinants of the regioselectivity possessed by TcPAM and paves the way for its biocatalytic applications on phenylalanine derivatives.

## 1 Introduction

**FIGURE 1:**
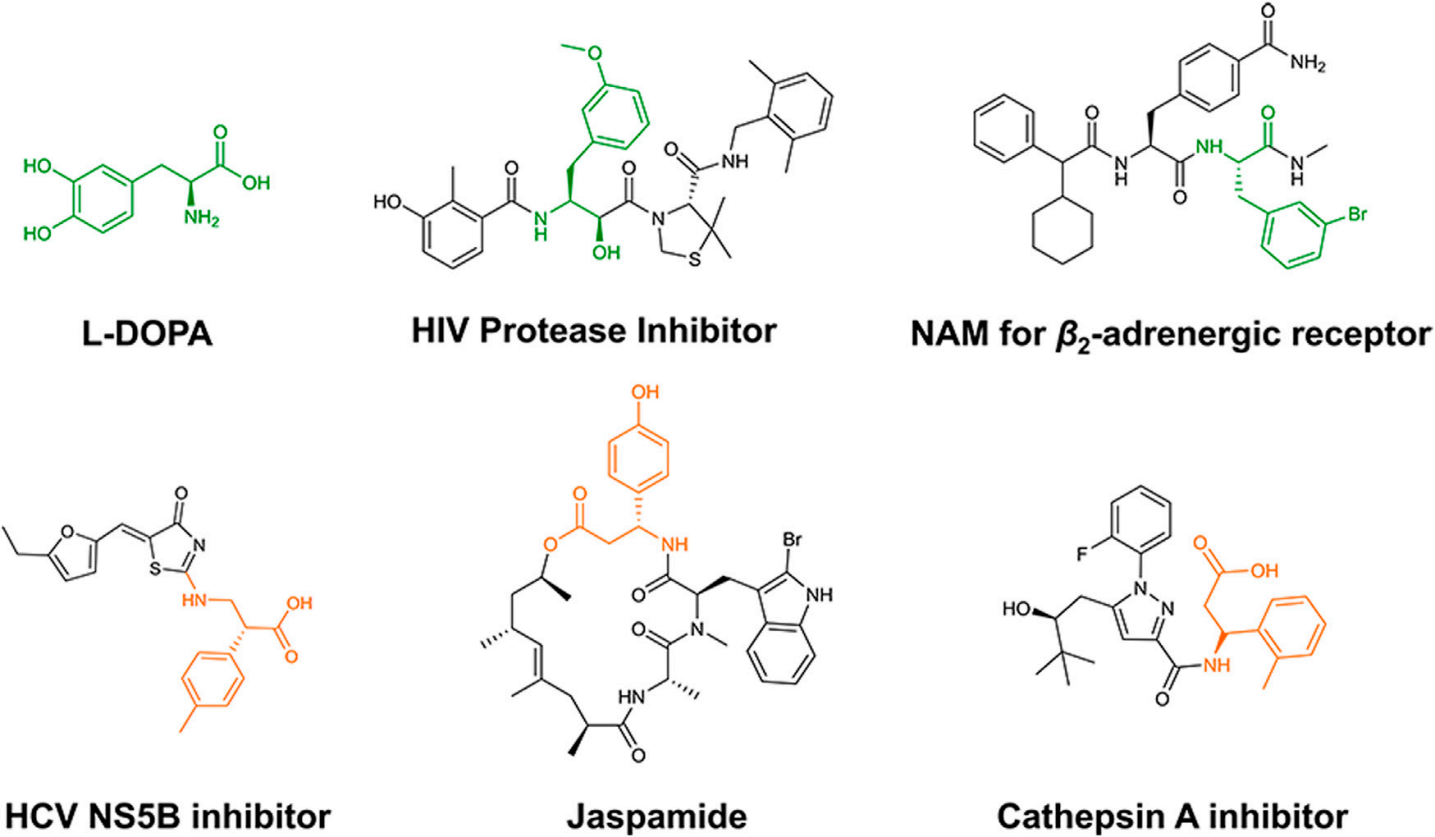
Representative bioactive compounds containing α- or β-phenylalanine derivatives. The α- and β-aryl alanine moieties are indicated in green and orange, respectively.

α*-* and β-phenylalanine derivatives are valuable building blocks in a variety of bioactive compounds (Xue et al., 2018; Parmeggiani et al., 2018; Wu et al., 2021). As shown in Figure 1, either α- or β-phenylalanine derivatives are important fragments in pharmaceuticals and bioactive compounds (Cotzias et al., 1969; Mimoto et al., 2004; Meng et al., 2018; Yan et al., 2007; Zabriskie et al., 1986), which are essential for the synthesis of these compounds. However, chemical syntheses with harsh conditions have hindered their preparations (Danthi and Hill, 1997; Chien et al., 2018; Guo et al., 2020).

Phenylalanine aminomutase (PAM) is an enzyme in the class I lyase-like family with a characteristic post-translational intrinsic cofactor 5-methylidene-imidazole-4-one (MIO), which functions as an electrophile for the two-step process: first, the initial de-amination of α-Phe to form *trans*-cinnamic acid (*t*-CA) and then the subsequent re-amination to transfer the amino group to either the α- or β-position for the generation of α- or β-Phe (Wu et al., 2009). When *t*-CA derivatives are served as substrates for the step of amination, enzymatic synthesis of α- or β-phenylalanine derivatives by PAM is a feasible approach, especially with the unique features of 100% theoretical atomic economy, easier accessibility, inexpensive substrates, and no requirement for additional cofactor(s). However, mainly due to the inherited poor regioselectivity of PAMs, the products resulting from the amination are usually a mixture containing an approximately equal amount of α- and β-isomers, and further purification is difficult and tedious. Among different sources, the PAM from *Taxus chinensis* (TcPAM) naturally catalyzes the interconversion between (*S*)-α-Phe and (*R*)-β-Phe during the biosynthesis of the *N*-benzoyl-2-phenyl-isoserine side chain of the anticancer drug Taxol, which has attracted significant attention for its applications in the biosynthesis of unnatural amino acids (Steele et al., 2005; Feng et al., 2011; Wybenga et al., 2014).

Commonly, the selectivity of enzymes toward unnatural substrates is one of the most important factors that decide their values as biocatalysts. Despite numerous reports on enzyme engineering for various changes in biocatalytic scope based on 3D structures and reaction mechanisms (Wu et al., 2021; Winkler et al., 2021), successful examples of improving regioselectivity are relatively few, which appears to be a difficult task to accomplish. Therefore, the elucidation of the residues that affect the regioselectivity could facilitate the engineering of this valuable enzyme for various purposes. Previously, significant efforts have been made in the engineering of TcPAM, and several important residues, such as L108, N458, Q319, and R325, have been identified and confirmed to impact the regioselectivity (Zhu et al., 2018; Wu et al., 2012). If the binding pocket of TcPAM is divided into two portions as carboxyl- and aromatic-binding sites, previous engineering efforts mainly focused on the carboxyl-binding site with the substrates of phenylalanine or cinnamic acid. Meanwhile, when substituted cinnamates were used as substrates, the regioselectivity was closely associated with the nature of the substitutions on the phenyl ring, resulting in various α- or β-selectivities, which were attributed to the positioning of the carboxylate-binding site (Wu et al., 2012; Szymanski et al., 2009). On the other hand, the regioselectivity variations by different substituted cinnamates implied that the aromatic-binding site might also be involved in the determination of regioselectivity. Thus, we speculated that the organization of the entire substrate-binding pocket of TcPAM is a decisive factor that determines the regioselectivity of cinnamate and its derivatives, and therefore, reconstructing the whole binding pocket could be a feasible approach to probe or improve its regioselectivity.

In this study, we identified new and critical residues in the aromatic-binding site that influence the regioselectivity of TcPAM for cinnamate and its derivatives. The reconstruction of the whole binding pocket and the synergistic changes in both carboxyl- and aromatic-binding sites resulted in improved outcomes in regioselectivity (Figure 3). The present study demonstrated the feasibility of reconstructing the binding pocket with minimized efforts in generating a smaller number of variants for regioselectivity improvement and provided a practical tool for the preparations of α- or β-phenylalanine derivatives.

## 2 Materials and methods

### 2.1 Materials

The commercial chemicals, analytical reagents, and solvents were obtained from Sigma-Aldrich Co. (Shanghai, China), McLean Co. (Shanghai, China), or Sinopharm (Shanghai, China) and used without further purification. The materials used for the medium were products of Oxoid (Shanghai, China) or Sinopharm (Shanghai, China). All biological materials for the molecular biology experiment were purchased from Takara (Beijing, China) or Beyotime (Hangzhou, China). The codon-optimized *tcpam* gene and primers used for mutagenesis were synthesized by Tsingke (Beijing, China).

### 2.2 Molecular docking

The Molecular Operating Environment (MOE) was used for molecular docking and ligand–enzyme interaction analysis (Vilar et al., 2008). Ligand *t*-CA was subjected to MOE for molecular preparation and energy minimization with standard default parameter settings. The intact tetramer structures of TcPAM (PDB ID: 3NZ4) (Feng et al., 2011) and the corresponding mutants were prepared using PyMOL (DeLano, 2002) and subsequently proceeded to 3D protonation and energy minimization using the force field of Amber10:EHT in MOE. The substrate was docked into the enzyme using the triangle matcher placement and induced fit refinement model. The top ranked docking poses for each ligand were generated for further substrate–enzyme interaction analysis in terms of docking score and binding energy. The PyMOL educational version was applied for the initial docking analysis and image processing.

### 2.3 Site-directed mutagenesis

All point mutations of TcPAM were generated from the codon-optimized wild-type *tcpam* gene (GenBank: AY582743.1) (Szymanski et al., 2009) in a pRSFDuet vector with the restriction enzyme sites of BamHI and HindIII and performed using PCR. Mutagenesis was carried out using QuickMutation™ (Beyotime, Hangzhou, China). DpnI digestion was conducted by adding 1 μL DpnI to PCR reaction mixtures and maintained at 37°C for 1 h to digest the methylated plasmid template. The primers used for mutagenesis are given in Supplementary Table 1.

### 2.4 Cloning, expression, and purification of TcPAM

The pRSFDuet expression vector containing codon-optimized TcPAM or TcPAM mutants and the plasmid pTf16 with a molecular chaperone were co-transformed to *E. coli* BL21 (DE3), and a single colony transformant was selected for inoculating to 5 mL of LB medium supplemented with 50 μg/mL kanamycin and 34 μg/mL chloramphenicol. After overnight growth at 37°C, the cultures were inoculated into 200 mL LB medium containing 50 μg/mL kanamycin and 34 μg/mL chloramphenicol. The cells continued to grow at 37°C and 220 rpm until reaching OD_600_ of 0.8∼1.0, at which the cultures were induced for protein expression by the addition of 0.1 mM IPTG at 16°C for 20 h. The cells were harvested by centrifugation (8,000 rpm, 10 min, 4°C), and cell pellets were collected for SDS-PAGE analysis and protein purification or stored at −80°C until further use.

TcPAM and its mutants were purified according to a previous report with modifications (Feng et al., 2011). The elution fractions generated from Ni-NTA affinity chromatography were desalted using a PD-10 column (GE HealthCare, Sweden) and subsequently concentrated using a 100 kDa Amicon Ultra-15 Centrifugal Filter Unit (Millipore, United States). The concentrated protein solution was purified using an anion exchange column (HiTrap Q HP from GE HealthCare) connected to the ÄKTA purification system, and a stepwise gradient of buffer A (50 mM Na_2_HPO_4_–NaH_2_PO_4_ and 5% glycerol, pH 8.0) and buffer B (50 mM Na_2_HPO_4_–NaH_2_PO_4_, 1 M NaCl, and 5% glycerol, pH 8.0) was applied at a gradient of 0%–100% buffer B with a column volume of 25 mL at a flow rate of 1.0 mL/min. The elution fractions containing TcPAM or mutants were combined and concentrated for further gel filtration chromatography (HiLoad 16/600 Superdex 200 pg column, GE HealthCare). The purity of the purified proteins was assessed by SDS-PAGE with Coomassie blue staining, and the quantity was determined by the Bradford protein assay.

### 2.5 HPLC analysis

Analytical HPLC was performed by gradient elution with solvent A (water containing 0.1% TFA) and solvent B (acetonitrile containing 0.1% TFA) at 40°C with UV detection at 210 nm and a flow rate of 1.0 mL/min under the following gradient: 5%–85% solvent B over 20 min.

Chiral HPLC was operated on a CHIRALPAK^®^ ZWIX (+) column (150 × 4.0 mm) by isocratic elution. The mobile phase consisted of methanol–acetonitrile–water (49:49:2, V/V/V) containing 100 mM formic acid and 25 mM diethylamine. Elution was achieved with a flow rate of 0.4 mL/min at 25°C by monitoring at 254 nm.

### 2.6 The optimization of the amination activity

The amination reaction system was optimized at a scale of 200 μL under varied conditions: five ammonium donors (0.5 M (NH_4_)_2_SO_4_, 1 M NH_4_Cl, 1 M NH_4_OAC, 1 M NH_4_OH, and 1 M NH_4_NO_3_), concentration of ammonia solution (1–8 M), pH (8.5–11), and temperature (25°C–70°C). The reactions were performed using 2 mM *t*-CA and 0.5 mg/mL TcPAM enzyme for 6 h. The reactions were terminated by boiling in a water bath for 10 min, and the supernatants were obtained by centrifugation for HPLC analysis. One unit (U) of enzyme activity was defined as the amount of enzyme that produced 1 μmol of product per minute under the described conditions.

### 2.7 Enzyme activity assay and kinetic parameters of TcPAM and its variants

The amination condition of *t*-CA and its derivatives was obtained by adding 2.5 mM substrate, 0.5 mg/mL TcPAM enzyme, and 6 M NH_4_OH (pH 10) to a volume of 200 μL at 35°C for 16 h. The reactions were terminated by boiling in a water bath for 10 min, and the supernatants were obtained by centrifugation for HPLC analysis. The conversion and product ratio were determined by analytical HPLC.

The kinetic determination of TcPAM and its mutants with the L-Phe substrate was conducted as follows: the enzyme at a final concentration of 0.2 mg/mL was incubated with L-Phe (0.1–8 mM) in 50 mM phosphoric acid buffer (pH 8.5) at 30°C for 15 min. After the termination of the reactions by boiling in a water bath for 10 min, the yields of β-Phe and *t*-CA were quantified by HPLC, and the reaction rates of various concentrations of L-Phe were calculated using the Michaelis–Menten equation.

The kinetic determination of TcPAM and its mutants with *t*-CA substrate was performed as follows: the enzyme at a final concentration of 0.5 mg/mL was incubated with *t*-CA (0.1–12 mM) in 6 M NH_4_OH at pH 10 and temperature 35°C for 30 min. After the termination of the reactions by boiling in a water bath for 10 min, the yields of α-Phe and β-Phe were detected by HPLC, and the reaction rates of various concentrations of *t*-CA were calculated using the Michaelis–Menten equation.

### 2.8 Amination reactions of substituted phenylalanine derivatives at a preparative scale

The reactions were conducted at a 100-mL scale. The purified mutants of L104A and Q319M were suspended in 2 M NH_4_OH buffer (pH 10.0, 10% glycerol) containing 6 mM *ortho*-chloro-cinnamic acid and 4 mM *para*-methyl-cinnamic acid, respectively. The reactions were conducted at 80 rpm at 35°C, and time courses were carried out by taking samples of 500 μL at different time points to monitor conversion rates and regioselectivity. The samples were quenched by heating for 10 min, and the products were further analyzed by analytical HPLC.

## 3 Results and discussion

### 3.1 Increase in the soluble expression and stability of TcPAM

**FIGURE 2:**
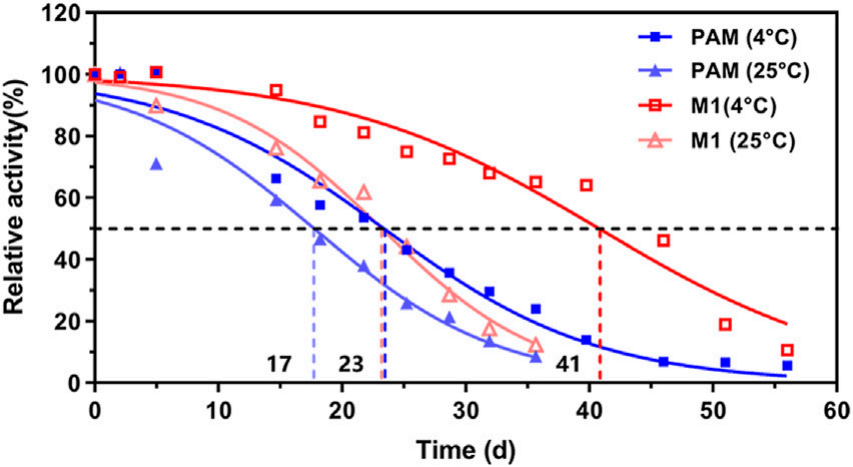
Effects of temperature on the stability of wild-type TcPAM and surface-engineered mutant M1 (C672S/C683S)^a^. ^a^WT TcPAM and M1 were stored in 50 mM phosphate buffer (pH 8.5, 10% glycerol) at 4°C and 25°C, respectively, at a final concentration of 4 mg/mL. The time courses of thermal stability were determined using 2 mM L-Phe and 0.2 mg/mL enzyme under a reaction condition of 50 mM phosphate buffer (pH 8.5) at 30°C.

The cysteine residues on the PAM surface are exposed to solvent, and the oxidations of these residues can easily result in cross-linking for protein aggregation during the expression or purification. According to the successful elimination of the aggregation issue by mutating the surface cysteines to serines in PALs (Wang et al., 2008), we first constructed a C672S/C683S (M1) variant of TcPAM and co-expressed with a molecular chaperone trigger factor (Hoffmann et al., 2010), resulting in the enhancement of soluble expression from ∼2.3 mg/L to ∼26.5 mg/L (Supplementary Figure 1). After the purification of M1 (Supplementary Figure 2), the thermal stability of this variant was also significantly improved with the extension of its half-life from 23.4 days to 40.7 days at 4°C (Figure 2). Further kinetic and stereochemical comparisons of wild-type (WT) TcPAM and M1 mutant toward L-Phe or *t*-CA revealed that the C672S/C683S mutant did not alter the catalytic property of TcPAM (Supplementary Table 2; Supplementary Figure 3). Thus, a thermostable enzyme with a high expression level and the same activity was obtained for subsequent enzyme engineering and amination comparison.

### 3.2 Identification of residues in the substrate-binding pocket to influence regioselectivity

**FIGURE 3:**
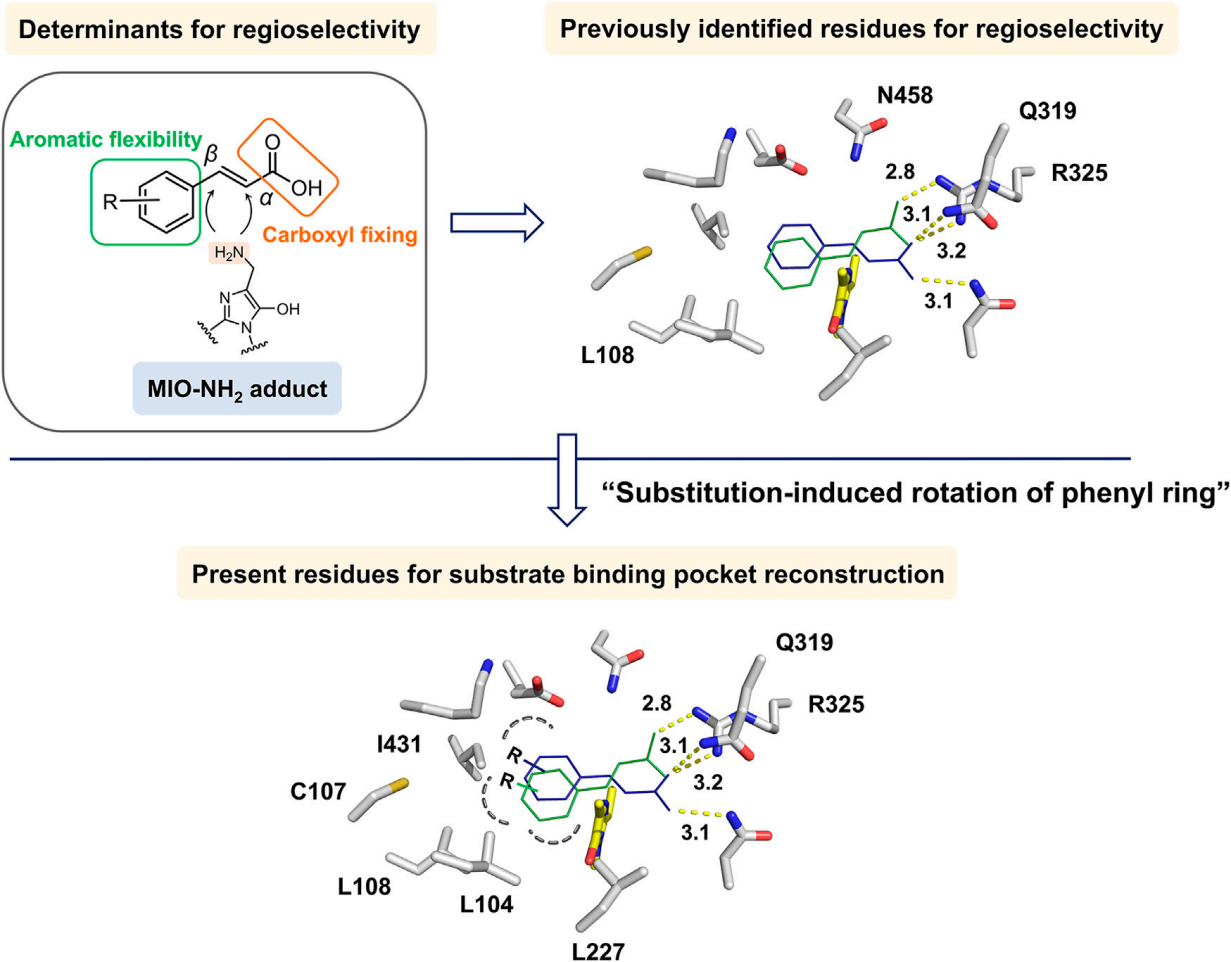
Identification of regioselectivity determinants of TcPAM in this study. The substrate *t*-CA was docked into WT TcPAM to result in two binding modes exposed to MIO. The reported and proposed residues critical to regioselectivity in aromatic- and carboxylate-binding sites are indicated. MIO is indicated in yellow.

Due to the presence of the intrinsic MIO moiety, the *de novo* creation of a complex structure of TcPAM with *t*-CA was not feasible. To establish a structural model for the analysis of the interactions, molecular docking of TcPAM (PDB ID: 3NZ4) (Feng et al., 2011) with *t*-CA was performed. The docking results indicated *t*-CA positions in the active site, leading to two substrate-binding modes facing MIO in opposite directions (Figure 3). Additionally, in the structural models, the phenyl ring stacks into a hydrophobic pocket for aromatic binding, and the carboxylate group electrostatically interacts with polar residues on the other side, which is similar to the previous binding model of TcPAM (Wybenga et al., 2014). Notably, Q319M and R325K mutants in the carboxylate-binding site were reported to be solely responsible for β-selectivity (Wu et al., 2012). However, previous studies on the modification of the hydrophobic-binding pocket of PALs showed better performance toward substituted *t*-CA derivatives (Nagy et al., 2019; Ahmed et al., 2018; Tork et al., 2022), suggesting that the aromatic-binding site also plays an important role, especially in the form of hydrophobic interactions and steric hindrance, in deciding the regioselectivity. Subsequently, according to the catalytic mechanism of TcPAM, the structure–function relationships of MIO-dependent enzymes, and prior efforts, we reasoned that the following considerations could be pivotal for the regioselectivity of TcPAM: 1) the regioselectivity might largely be dependent upon the flexibility of the “*t*-CA rotamer” in the active site, and the decreased steric hindrance of the aromatic-binding site might alter the orientation for the regioselective accommodation of *t*-CA and its derivatives; 2) As previously reported (Wu et al., 2012), the residues of Q319 and R325 located in the carboxylate-binding site contributed to the regioselectivity; 3) The hydrophobicity and size of the residues in the aromatic-binding site are critical for the regioselectivity. Therefore, simultaneous changes in both aromatic- and carboxylate-binding sites to reconstruct the whole substrate-binding pocket might be necessary to significantly affect the regioselectivity.

First, to identify the residues that potentially influence the regioselectivity, we selected five hydrophobic residues in the aromatic-binding site for mutagenesis with the substrate of *t*-CA. The residues of L104, L108, L227, and I431 were individually mutated to Ala or Val for the enlargement of the binding pocket, and the C107 residue responsible for substrate recognition (Wu et al., 2010) was replaced by Ser, Ala, or Val. Given the previous mutagenesis results on residues Q319 and R325 and their important roles for *t*-CA positioning, the R325K mutant was constructed to disrupt the formation of a salt bridge, and Q319 was mutated to Glu that is presented in homologous proteins EncP (Weise et al., 2015) and Leu or Met. As a result, 15 mutants were generated using M1 as the template.

**FIGURE 4:**
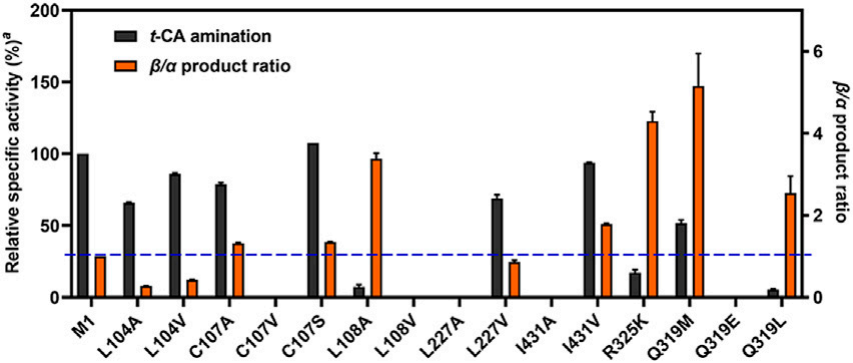
Comparison of *t*-CA amination activity and β-selectivity^b^. M1 was used as the template for mutagenesis; the blue dotted line represents the β/α product ratio of M1. ^a^Relative to M1. ^b^Reaction conditions: enzyme (0.5 mg/mL), *t*-CA (2.5 mM), and NH_4_OH (6 M, pH 10) in a volume of 200 μL at 35°C for 16 h.

Next, under optimal conditions (Supplementary Figures 6–9), when *t*-CA was served as the substrate for amination, the expansion of the aromatic-binding site generally maintained the amination activity compared to M1 (Figure 4), among which mutant C107S exhibited a slightly enhanced activity and β-selectivity. Meanwhile, mutant L104A showed the greatest α-selectivity for *t*-CA to yield 78% of α-Phe, while L108A displayed 77% β-selectivity, demonstrating the importance of steric effect in the aromatic-binding site for regioselectivity. Previously, mutant L104A significantly influenced the product ratio for the natural substrate of L-Phe, leading to a preference for *t*-CA formation over β-Phe (Feng et al., 2011). Presently, we found that α-Phe was the dominant product yielded by L104A from its amination. The opposite regioselectivity, α- vs. β-amination product ratios of 69:31 and 35:65, by L104V and I431V, suggested that the hydrophobic interaction might be involved in the binding process with *t*-CA. When I431 was mutated to Ala, the amination activity was completely abolished, indicating that appropriate steric hindrance and hydrophobic interaction are required to stabilize the phenyl ring and assist the positioning of the carboxyl group. According to a previous report (Wu et al., 2012), the β-selectivity of TcPAM could be facilitated by the mutations in the carboxyl-binding site, and the charge density (R325K) or steric hindrance (Q319M) might be helpful to the increase in β-addition. In the present study, the results of R325K, Q319M, and Q319L confirmed their favorable β-selectivity (71%∼84%), whereas their catalytic efficiency was decreased compared to M1 (Supplementary Table 3). Meanwhile, the combined mutagenesis of Q319M/R325K at the carboxyl-binding site resulted in the complete loss of activity (Wu et al., 2012), suggesting that solely engineering such a binding site cannot achieve further improvement of the regioselectivity.

Collectively, the comparison of regioselectivity toward substrate *t*-CA from the mutagenesis enabled us to identify several new residues related to regioselectivity in the aromatic-binding site, such as L104, C107, L227, and I431.

**FIGURE 5:**
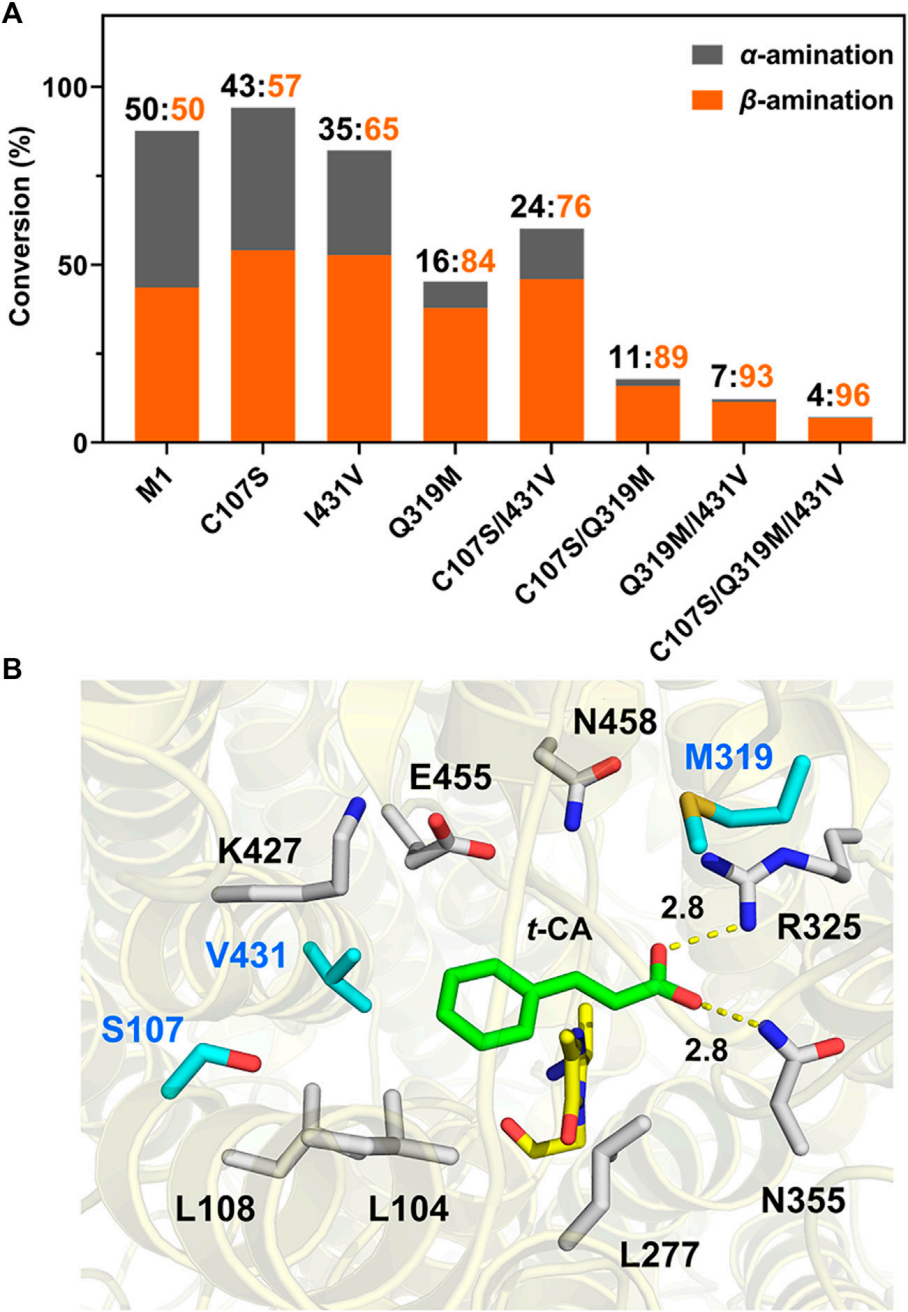
Regioselectivity of mutagenesis and possible molecular basis^a^. **(A)** Comparison of regioselectivity of single or combined mutagenesis for *t*-CA amination activity and β-selectivity. **(B)** Docking results of mutant M5 with the *t*-CA molecule. Mutated residues in the substrate-binding pocket are marked in blue, and the interactions between carboxylate groups of the substrate with the carboxyl-binding site were measured and are labeled. MIO is indicated in yellow. ^a^Reaction conditions: enzyme (0.5 mg/mL), *t*-CA (2.5 mM), and NH_4_OH (6 M, pH 10) in a volume of 200 μL at 35°C for 16 h.

To further confirm the importance of these residues in the regioselectivity in a cooperative manner, combined mutagenesis was subsequently performed based on the higher activity of C107S and I431V in the aromatic-binding site and better β-selectivity of Q319M in the carboxylate-binding site. As a result, C107S/I431V (M2), C107S/Q319M (M3), Q319M/I431V (M4), and C107S/Q319M/I431V (M5) were generated and compared. Among these, variant M5 showed β-selectivity of 96% toward *t*-CA and a 25.5-fold increase in the β/α product ratio compared to M1 (Figure 5). Moreover, we constructed additional mutants to further support the cooperative fashion on the regioselectivity (Supplementary Figure 10). The results strongly suggested that despite the decrease in amination activity, the aromatic- and carboxylate-binding sites cooperatively affect the regioselectivity.

Subsequently, to provide a reasonable explanation for the exceptional regioselectivity of variant M5, molecular docking was performed to establish a structural basis based on the availability of crystal structures of TcPAM complexed with *t*-CA. As shown in Figure 5, *t*-CA is well fitted in the reconstructed substrate-binding pocket. The enlarged aromatic-binding pocket by mutating Ile to Val at position 431 may facilitate the cinnamate skeleton to function as a “*t*-CA rotamer” for exposing C_β_ to MIO at the Re-face for β-addition, which is in accordance with the stereochemistry of TcPAM as an (*R*)-selective enzyme (Wybenga et al., 2014). More importantly, the mutations in the carboxyl-binding site, particularly Q319M, might cooperatively stabilize such a binding mode for convenient β-amination. Additionally, since the increase in regioselectivity of TcPAM from mutagenesis was generally accompanied by a decrease in activity, the C107S mutant was likely to compensate for such a trade-off between regioselectivity and activity, which could be a point for further enzyme engineering.

### 3.3 Confirmation of the regioselectivity changes in the reconstructed binding pocket by *t*-CA derivatives

To verify that the whole substrate-binding pocket is essential for regioselectivity, phenyl-substituted substrates were used as probes for the examination of mutants with the changes in regioselectivity, which could resemble the pocket reconstruction for confirmation.

**FIGURE 6:**
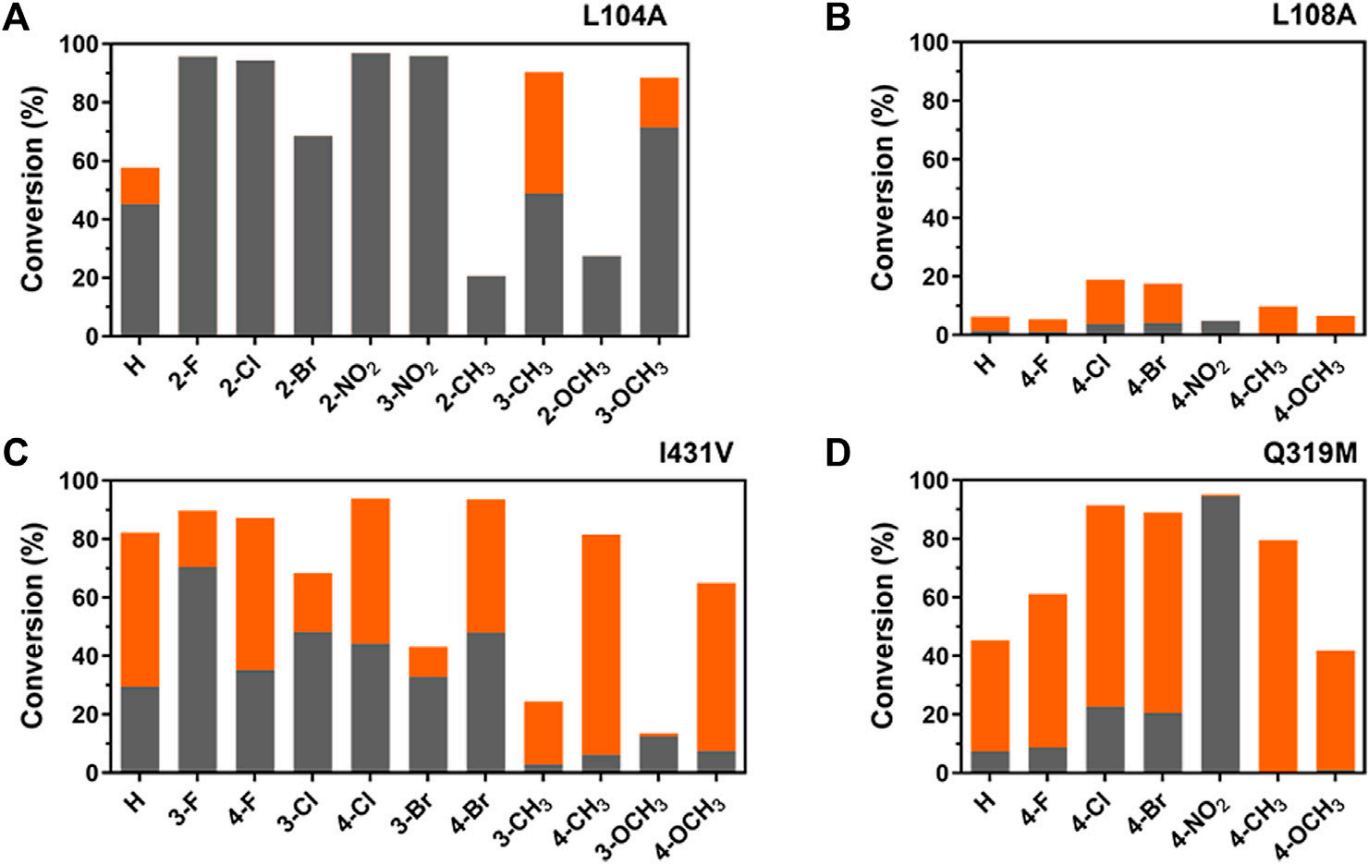
Comparative illustration of regioselectivity and activity of TcPAM mutants for the amination of *t*-CA derivatives^a^. Positions 2, 3, and 4 refer to *ortho*-, *meta*-, and *para*-substitutions on the phenyl ring. **(A)** Product distribution of L104A-catalyzed amination reactions; **(B)** product distribution of L108A-catalyzed aminations; **(C)** product distribution of I431V-catalyzed aminations; and **(D)** product distribution of Q319M-catalyzed aminations. Gray and orange bars represent the proportions of α-products and β-products, respectively. ^a^Reaction conditions: enzyme (0.5 mg/mL), substrate (2.5 mM), and NH_4_OH (6 M, pH 10) in a volume of 200 μL at 35°C for 16 h.

As shown in Figure 6, regioselectivity variations were indeed more obvious between mutants and substitutions than the substrate of *t*-CA, further supporting the connectivity between aromatic- and carboxylate-binding sites in the regioselectivity. Previously, it was shown that WT TcPAM cannot accept ortho-methoxy-substituted *t*-CA as a substrate (Szymanski et al., 2009); however, reducing unfavorable steric clash in the aromatic-binding site by Leu to Ala at position 104, 100% α-selectivity was observed in the present study. Meanwhile, the regioselectivity for *meta*-methyl-substituted t-CA changed from 80% β-selectivity to 54% α-selectivity by the L104A mutant, suggesting that a spatial orientation of residue 104 toward the phenyl ring is preferred for a certain degree of regioselectivity. Additionally, L108A and I431V mutants exhibited different regioselectivity levels toward *para*- or *meta*-substituted substrates, indicating that the microenvironment of the aromatic binding site is also crucial for the regioselectivity.

Regarding *para*-substituted substrates, mutant Q319M showed the desired regioselectivity, either α- or β-selectivity, depending on the substitutions, which is similar to previous results (Wu et al., 2012). Although the positioning of the substrates by the carboxylate-binding site is important for regioselectivity, the combination of aromatic- and carboxylate-binding sites as a whole is even more critical to determining the final outcomes on the regioselectivity. Therefore, comprehensive engineering of the entire binding pocket of TcPAM should be a necessity for improving the regioselectivity.

### 3.4 Regioselectivity of desired variants at a preparative scale

**FIGURE 7:**
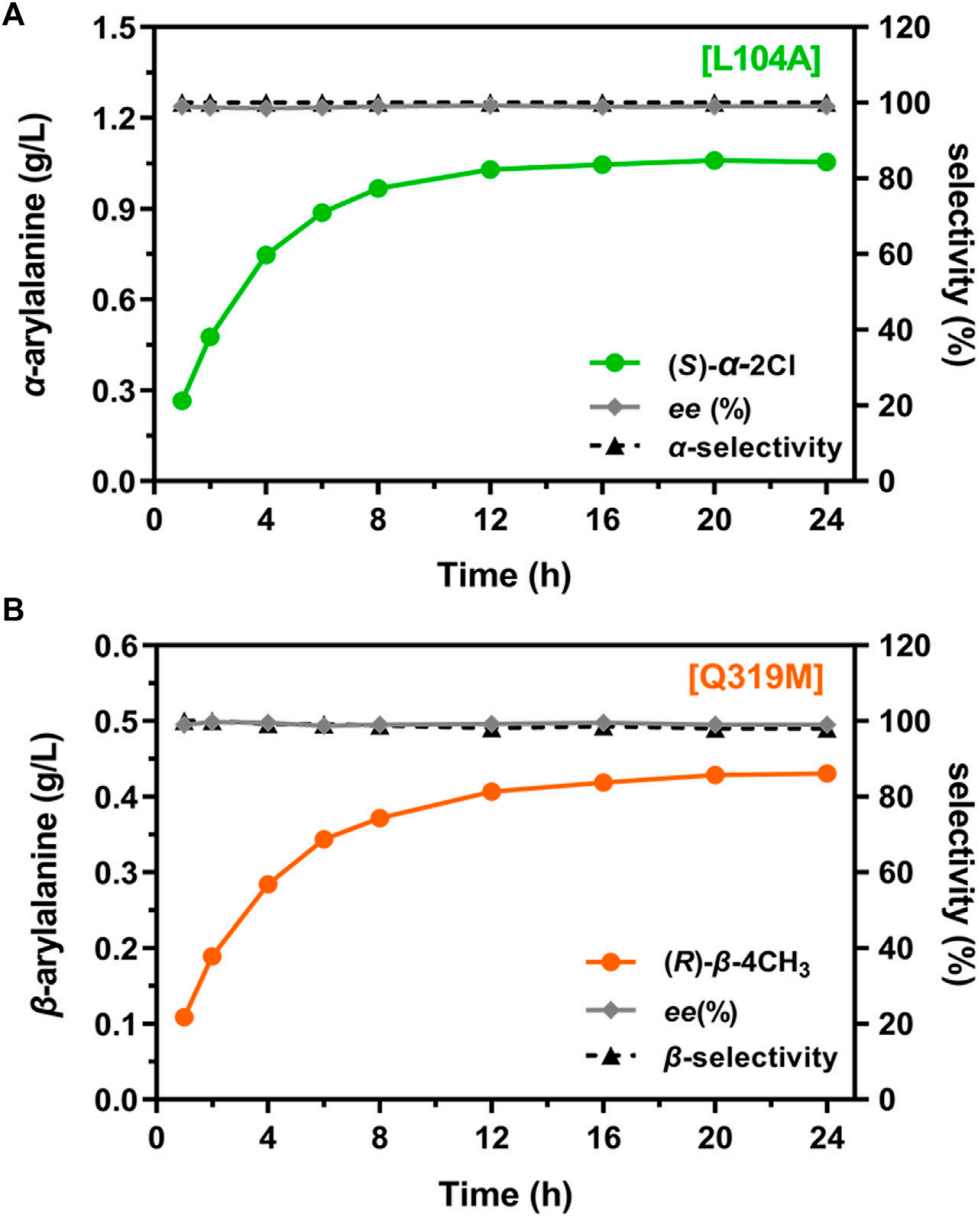
Regioselectivity of the mutants for aminations at a preparative scale. **(A)** L104A-catalyzed amination^a^; **(B)** Q319M-catalyzed animation^b^. Product concentration, regioselectivity, and enantiomeric excess for 2-chloro-α-Phe and 4-methyl-β-Phe are indicated. ^a^Reaction conditions: enzyme (0.5 mg/mL), substrate (6 mM), and NH_4_OH (2 M, pH 10) in a volume of 100 mL at 35°C for 24 h. ^b^Reaction conditions: enzyme (1 mg/mL), substrate (4 mM), and NH_4_OH (2 M, pH 10) in a volume of 100 mL at 35°C for 24 h.

To further examine the regioselectivity at a preparative scale, L104A and Q319M were selected for the *regio*-selective synthesis of two valuable precursors of 2-chloro-α-Phe (Jianke et al., 2012) and 4-methyl-β-Phe (Yan et al., 2007), respectively, at a 100-mL scale. After 16 h of reactions, both α-amination and β-amination reached a stationary phase with a product concentration of 1.05 g/L and 0.43 g/L, respectively (Figure 7). More importantly, high regioselectivity [α_(L104A)_ = 100% and β_(Q319M)_ = 98%] and enantioselectivity [*ee*_(L104A)_ > 99% and *ee*_(Q319M)_ > 99%] (Supplementary Figures 4, 5) were obtained at this scale, confirming a bidirectional synthetic utility of TcPAM engineering for the preparation of value-added α- and β-Phe derivatives.

## 4 Conclusion

In summary, we identified the determinants that affect the regioselectivity of TcPAM. The newly identified residues in the aromatic-binding site were confirmed to be critical for regioselectivity toward *trans*-cinnamate and its substituted derivatives. In addition, the connectivity of the aromatic-binding site and carboxylate-binding site in the substrate-binding pocket in determining the regioselectivity was demonstrated to be crucial. Furthermore, the variants of TcPAM with excellent regioselectivity were verified at a preparative scale for the preparation of phenylalanine derivatives.

To the best of our knowledge, we achieved the highest β-selectivity of TcPAM for the amination of *t*-CA. The implementation of a semi-rational strategy in this study successfully accomplished the reconstruction of the binding pocket of TcPAM for regioselectivity improvement, offering feasibility for future biocatalytic applications.

## Data Availability

The datasets presented in this study can be found in online repositories. The names of the repository/repositories and accession number(s) can be found in the article/Supplementary Material.
