# Supplementary material for: Improving regioselectivity of phenylalanine aminomutase from Taxus chinensis by semi-rational mutagenesis for the biocatalytic amination of cinnamates
Source: Front Bioeng Biotechnol. 2024 Aug 22;12:1417962. doi: 10.3389/fbioe.2024.1417962 (PMC11374720; doi:10.3389/fbioe.2024.1417962)
Supplement: Supplementary file 1 [file DataSheet1.docx]

***Supplementary Material***

# Supplementary Tables

**Supplementary Table 1.** List of the primers used for site-directed mutagenesis.

| Primers | Sequence（5'→3'）of primers*^a^* |
| --- | --- |
| C672S-F  C672S-R  C683S-F  C683S-R  L104A-F | GGTTCCACTGCTGCAT**AGT**CTGCAAGGCTTTCTGG  CCAGAAAGCCTTGCAG**ACT**ATGCAGCAGTGGAACC  GGTCAGCCAAACGGT**AGC**GCCAACGGCGTTGAG  CTCAACGCCGTTGGC**GCT**ACCGTTTGGCTGACC  CGAACTCCAAGAAAGT**GCC**ATCCGTTGTCTGCTG |
| L104A-R | CAGCAGACAACGGAT**GGC**ACTTTCTTGGAGTTCG |
| L104V-F | CGAACTCCAAGAAAGT**GTT**ATCCGTTGTCTGCTG |
| L104V-R | CAGCAGACAACGGAT**AAC**ACTTTCTTGGAGTTCG |
| C107A-F | AGAAAGTCTCATCCGT**GCC**CTGCTGGCCGGCGT |
| C107A-R | ACGCCGGCCAGCAG**GGC**ACGGATGAGACTTTCT |
| C107V-F | AGAAAGTCTCATCCGT**GTT**CTGCTGGCCGGCGT |
| C107V-R | ACGCCGGCCAGCAG**AAC**ACGGATGAGACTTTCT |
| C107S-F | AGAAAGTCTCATCCGT**AGC**CTGCTGGCCGGCGT |
| C107S-R | ACGCCGGCCAGCAG**GCT**ACGGATGAGACTTTCT |
| L108A-F | AAGTCTCATCCGTTGT**GCG**CTGGCCGGCGTGTT |
| L108A-R | AACACGCCGGCCAG**CGC**ACAACGGATGAGACTT |
| L108V-F | AAGTCTCATCCGTTGT**GTT**CTGGCCGGCGTGTT |
| L108V-R | AACACGCCGGCCAG**AAC**ACAACGGATGAGACTT |
| L227A-F | CAAGCCAAGGAAGGT**GCG**GCGCTGGTTAATGGC |
| L227A-R | GCCATTAACCAGCGC**CGC**ACCTTCCTTGGCTTG |
| L227V-F | CAAGCCAAGGAAGGT**GTT**GCGCTGGTTAATGGC |
| L227V-R | GCCATTAACCAGCGC**AAC**ACCTTCCTTGGCTTG |
| I431A-F | TCTGAAAGGCCTCGAC**GCG**GCGATGGCGGCCTAT |
| I431A-R | ATAGGCCGCCATCGC**CGC**GTCGAGGCCTTTCAGA |
| I431V-F | TCTGAAAGGCCTCGAC**GTT**GCGATGGCGGCCTAT |
| I431V-R | ATAGGCCGCCATCGC**AAC**GTCGAGGCCTTTCAGA |
| R325K-F | GATCGCTACGCGCTG**AAA**AGTAGTCCACAGTGG |
| R325K-R | CCACTGTGGACTACT**TTT**CAGCGCGTAGCGATC |
| Q319E-F | CTGAAGAAGCCGAAG**GAA**GATCGCTACGCGCTG |
| Q319E-R | CAGCGCGTAGCGATC**TTC**CTTCGGCTTCTTCAG |
| Q319M-F | CTGAAGAAGCCGAAG**ATG**GATCGCTACGCGCTG |
| Q319M-R | CAGCGCGTAGCGATC**CAT**CTTCGGCTTCTTCAG |
| Q319L-F | CTGAAGAAGCCGAAG**CTG**GATCGCTACGCGCTG |
| Q319L-R | CAGCGCGTAGCGATC**CAG**CTTCGGCTTCTTCAG |

^a^The codons for amino acids at the mutational sites are colored in red.

**Supplementary Table 2.** Kinetic parameters of wild type TcPAM (WT) and C672S/C683S mutant (M1).

| Subsrate | Enzyme | *K*_m_(mM) | *V*_max_ (U/mg)×10^3^ | *k*_cat_  (s^-1^)×10^3^ | *k*_cat_/*K*_m_  (M^-1^ s^-1^) |
| --- | --- | --- | --- | --- | --- |
| *L*-phe^a^ | WT | 0.37±0.02 | 174.6±2.1 | 218.8±2.6 | 592.3±25.0 |
|  | M1 | 0.46±0.02 | 231.2±2.7 | 289.8±3.4 | 630.6±20.0 |
| *t*-CA^b^ | WT | 3.10±0.4 | 46.4±2.0 | 58.1±2.5 | 18.9±1.6 |
|  | M1 | 3.70±0.3 | 59.6±1.7 | 74.7±2.1 | 20.2±1.1 |

^a^Reaction conditions: enzyme (0.2 mg/mL), *L*-phe (0.1-8 mM) in 200 μl phosphoric acid buffer (pH 8.5) at 30°C for 15 min.

^b^Reaction conditions: enzyme (0.5 mg/mL), *t*-CA (0.1-12 mM), NH_4_OH (6 M, pH 10) in 200 μl volume at 35°C for 30 min.

**Supplementary Table 3.** Kinetic parameters and *enantio*-selectivity of TcPAM mutants^a^.

| Enzyme | *K*_m_  (mM) | *k*_cat_  (s^-1^)×10^3^ | | *k*_cat_/*K*_m_  (M^-1^ s^-1^) | *ee* (β-Phe) | *ee* (α-Phe) | β : α |
| --- | --- | --- | --- | --- | --- | --- | --- |
| M1*^b^* | 3.7±0.3 | 74.7±2.1 | 20.2±1.1 | | > 99% | > 99% | 49:51 |
| L108A^c^ | 8.6±1.4 | 3.6±0.3 | 0.4±0.03 | | > 99% | > 99% | 77:23 |
| I431V*^c^* | 7.7±0.7 | 75.7±3.0 | 9.9±1.3 | | > 99% | > 99% | 64:36 |
| Q319M*^c^* | 0.8±0.1 | 12.1±0.4 | 15.1±2.0 | | > 99% | > 99% | 84:16 |
| R325K*^c^* | 4.2±0.8 | 12.9±0.6 | 3.1±0.5 | | > 99% | > 99% | 81:20 |

^a^Reaction conditions: enzyme (0.5 mg/mL), t-CA (0.1-12 mM), NH_4_OH (6 M, pH 10) in 200 μl volume at 35°C for 30 min.

^b^TcPAM (C672S/C683S) was used as initial enzyme (M1) for the reconstruction;

^c^Mutants were generated from the template of M1.

# Supplementary Figures


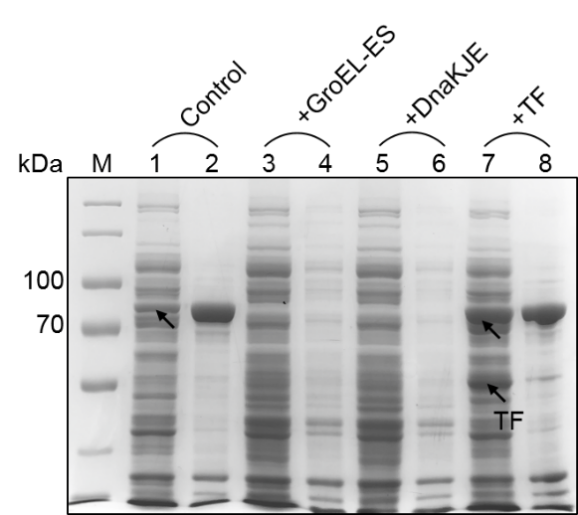


**Supplementary Figure 1.** Effects of molecular chaperones on the expression of M1. M: protein marker; Lane 1, 3, 5, 7: crude extract after induction; Lane 2, 4, 6, 8: pellet after induction. Control: M1 alone; GroES-EL: co-expression of M1 and GroES-GroEL; DnaKJE: co-expression of M1 and DnaK-DnaJ-GrpE; TF: co-expression of M1 and Trigger factor.


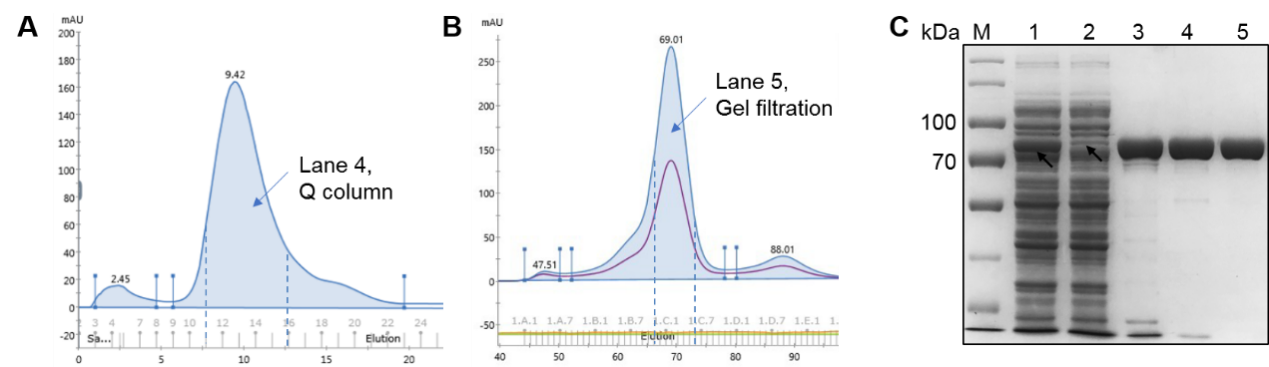


**Supplementary Figure 2.** Purification of M1. (A) Ion exchange chromatography; (B) Gel filtration chromatography; (C) SDS-PAGE analysis. M: protein marker; Lane 1: crude extract; Lane 2: flow through of Ni-affinity column; Lane 3: elute from Ni-affinity column; Lane 4: elute from Q column; Lane 5: elute from gel filtration.


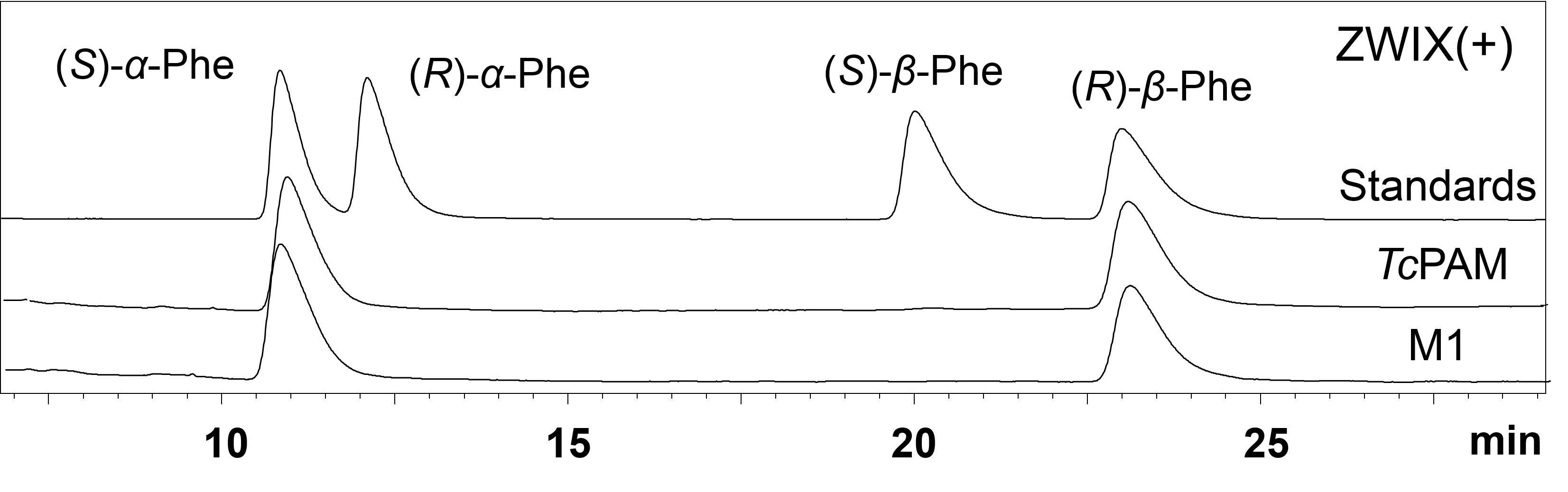


**Supplementary Figure 3.** HPLC chromatograms showing the separation of authentic standards of *regio*-isomers and enantiomers and the products generated by TcPAM- and M1-catalyzed reactions.


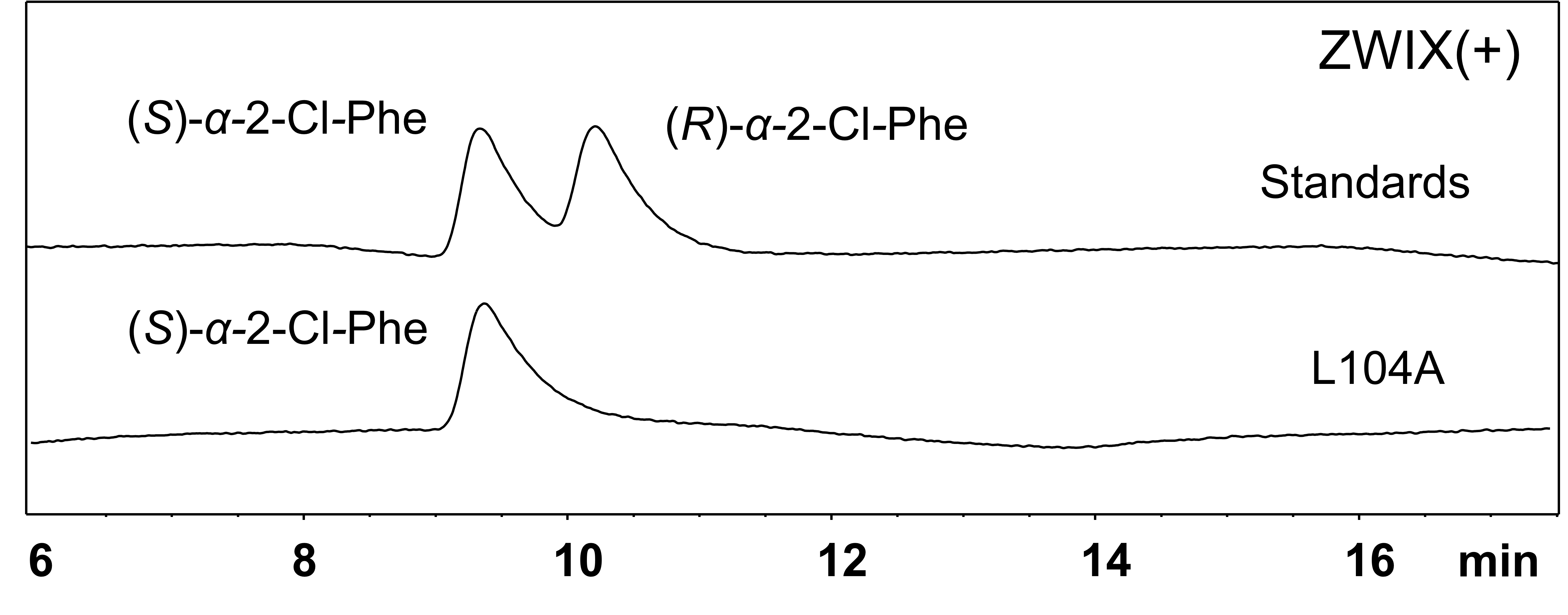


**Supplementary Figure 4.** HPLC chromatograms showing the separation of authentic standards of *regio*-isomers and enantiomers and the product generated by L104A-catalyzed reactions.


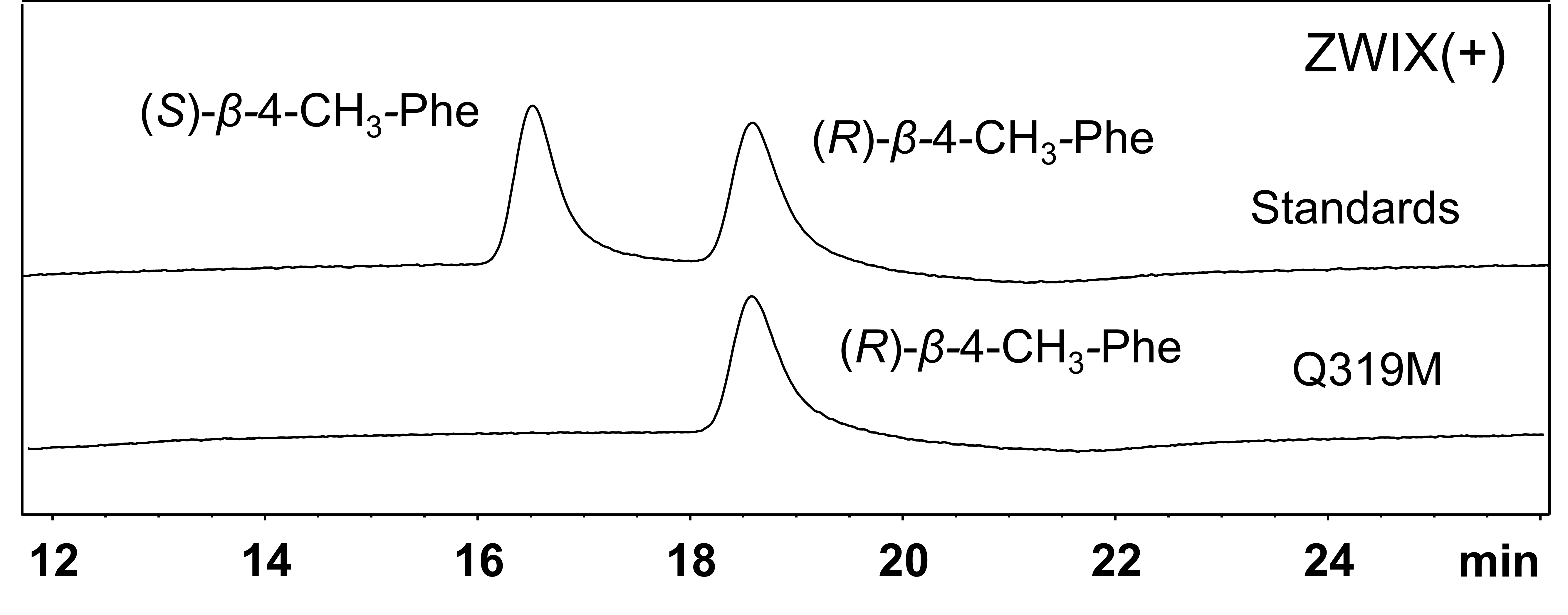


**Supplementary Figure 5.** HPLC chromatograms showing the separation of authentic standards of amino acid *regio*-isomers and enantiomers and the product generated by Q319M-catalyzed reactions.


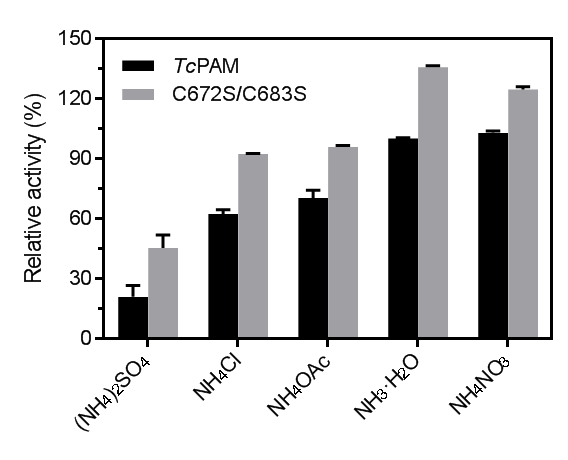


**Supplementary Figure 6.** Effects of ammonia donor on the amination activity of WT TcPAM and M1.


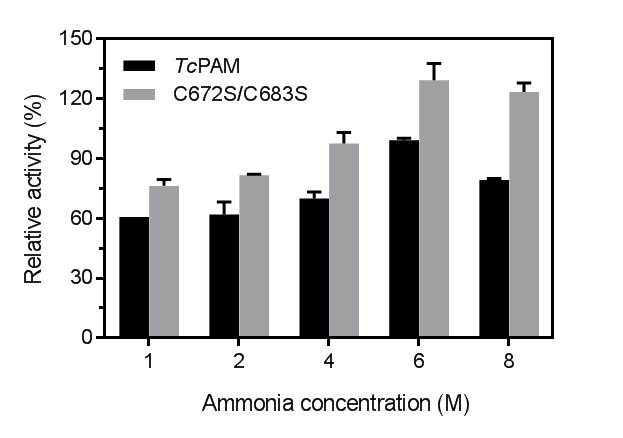


**Supplementary Figure 7.** Effects of ammonia solution concentration on the amination activity of WT TcPAM and M1.


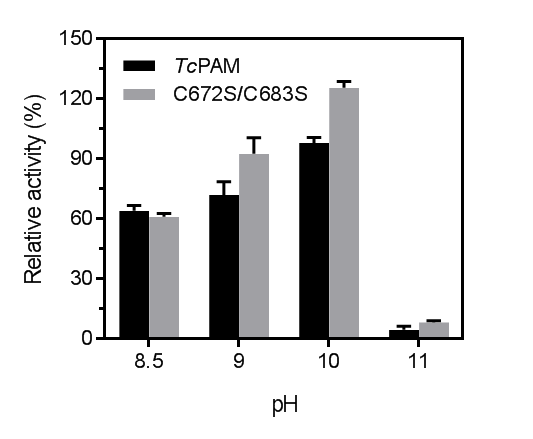


**Supplementary Figure 8.** Effects of pH on amination activity of WT TcPAM and M1.


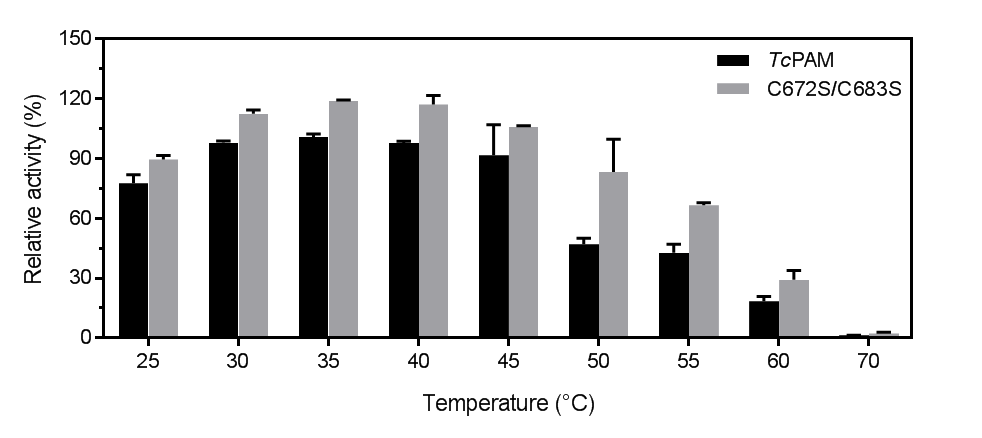


**Supplementary Figure 9.** Effects of temperature on amination activity of WT TcPAM and M1.


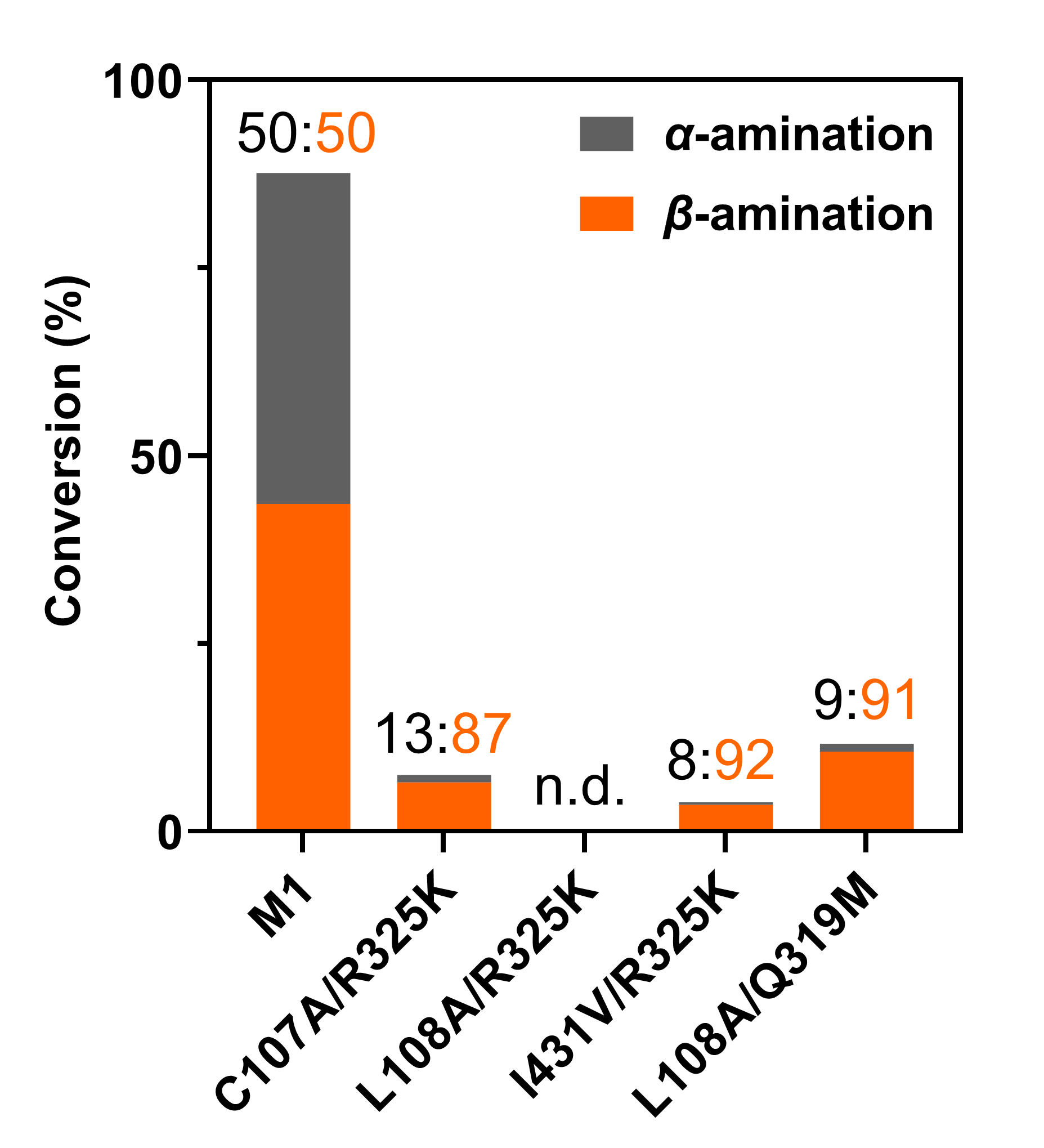


**Supplementary Figure 10.** Comparison of combined mutagenesis for *t*-CA amination activity and regioselectivity. n.d. indicates not detectable.
